# Exercise in Clinical Reasoning: Getting a Foothold on Lower Extremity Weakness

**DOI:** 10.1007/s11606-024-08624-x

**Published:** 2024-03-21

**Authors:** David Lester, Manisha Kotay, Qihua Fan, Gustave Weiland

**Affiliations:** 1 Virginia Commonwealth University School of Medicine, 1101 E. Marshall St, Box 980102, Richmond, VA 23298 USA; 2 Virginia Commonwealth University Health System, 1250 E. Marshall Street, Richmond, VA 23219 USA

In this series, a clinician extemporaneously discusses the diagnostic approach (regular text) to sequentially presented clinical information (bold). Additional commentary on the diagnostic reasoning process (italics) is integrated throughout the discussion.

**An 88-year-old man with hypertension presented to the emergency room with 6 weeks of progressive bilateral lower extremity weakness.**

The patient’s presenting issue of subacute, progressive bilateral lower extremity weakness has a broad differential. His history of hypertension, while a common chronic illness given his age, increases his risk for peripheral vascular disease and stroke. However, the progression and subacute nature deter from considering stroke higher on the differential as strokes generally are not progressive, but present with sudden and stable deficits.^1,2^ Unless the patient has generalized cerebral pathology, it would be unlikely to be bilateral.^3^ Alternatively, he may be experiencing a disease affecting the spinal cord (e.g., spinal cord compression, CNS lesion), peripheral nerves (e.g., B12 deficiency, diabetic polyneuropathy), neuromuscular junction (NMJ) (e.g., myasthenia gravis or Lambert Eaton syndrome), or muscle (e.g., polymyalgia rheumatica, myositis). Additional history and a physical exam will help localize the area of concern and better characterize his pattern of weakness.

*Problem representation is an early step in the process of clinical reasoning that helps highlight the important points of the case. Developing this representation early in the patient’s work up allows one to explore potential diagnostic frameworks (systematic approach to a clinical problem outside of the clinician’s experiences and expertise).*^*4*^* Problem representation uses semantic modifiers and descriptors that help define a patient’s illness. These terms include time course (acute, subacute, and chronic), illness course (progressive vs. stable), age (young vs. elderly), and risk factors (smoking, hypertension, diabetes, *etc*.). Obtaining more information will help develop and refine the problem representation thus helping the clinician develop an initial differential of the patient’s presenting problem and a framework of this problem to reduce cognitive load.*

**The** **patient developed mild, generalized weakness after a hospitalization for pneumonia a few months prior. After this hospitalization, he ambulated with a walker, but then developed difficulty rising from a chair. This progressed to being bed-bound and unable to lift his legs over 6 weeks. There was no fluctuation in weakness throughout the day. He was given a short course of prednisone several weeks prior to admission, but his symptoms did not improve.**

**He has no pain, incontinence, or sensory symptoms.**

**He has a history of hypertension and sick sinus syndrome with remote pacemaker placement. He takes metoprolol but was recently started on hydralazine for persistently elevated blood pressure during his recent hospitalization. He takes atorvastatin for primary prevention of stroke and myocardial infarction. He does not smoke or drink alcohol.**

**On exam,** **vital signs were within normal limits. On neurological exam, his pupils were round and equally reactive to light. Extraocular movement was intact. He had no ptosis. Passive range of motion was intact without rigidity. Strength was 3/5 in the bilateral lower extremities. Patellar and ankle reflexes were diminished but symmetric. Babinski reflex was not present. Sensation was intact throughout. There was no atrophy, tremor, fatiguability, or fasciculations. Tone was normal. Straight leg tests were negative. On cardiovascular exam, pulses were intact throughout. JVD was not present. Pitting edema was 2 + bilaterally in the lower extremities.**

Refining the problem representation using the modifiers obtained can help identify a framework and approach to diagnostic testing.

“An elderly man with a history of uncontrolled hypertension presented to the emergency room with subacute, progressive motor weakness of the proximal bilateral lower extremities.”

Utilizing a framework for lower extremity weakness can help diagnosis and treat the patient’s illness.^5^ Categorizing the exam findings within the nervous system should be performed.

Upper motor neurons (UMN) are in the cortex, brainstem, and corticospinal tracts. Diseases that involve upper motor neurons include stroke, multiple sclerosis, amyotrophic lateral sclerosis, and masses of the CNS. The patient’s lack of increased muscle tone, hyperreflexia, and Babinski sign (upper motor neuron signs) make upper motor neuron pathology unlikely.^2,3^

Lower motor neurons (LMN) are found in the anterior horn in the spinal cord, nerve roots, and peripheral nerves. LMN disease can be further subdivided into weakness that improves/worsens with repetition (acting at the neuromuscular junction), the presence of sensory deficits, or asymmetric vs. symmetric disease.

Regarding LMN pathology, a neuromuscular junction disorder such as myasthenia gravis (an autoimmune attack on post synaptic acetylcholine receptors) can cause proximal weakness, but typically shows a fatigable weakness which progresses throughout the day and often will have ocular or bulbar symptoms. The patient does not have fatigability or oculo-bulbar symptoms. Lambert Eaton syndrome, an autoimmune process against calcium channel receptors preventing presynaptic acetylcholine release, also can present with proximal weakness, but generally improves with use and often is associated with autonomic dysfunction. Another etiology includes an inflammatory demyelinating polyradiculoneuropathy such as Guillain-Barré syndrome (GBS), often characterized by acute, symmetric weakness with hyporeflexia that progresses over several weeks, typically nadiring by 4 weeks. His progression over 6 weeks, intact reflexes, and lack of sensory changes argues against GBS.^2^

The patient has no sensory loss, which lowers the possibility of peripheral neuropathies (diabetes, alcohol use, vitamin deficiencies, infection, and cancer induced) as etiologies.

*The clinician has reframed the patient’s problem representation and applied an established framework to approach the patient’s motor weakness by localizing the disease process. The clinician characterizes the patient’s weakness as a motor and not a sensory deficit as well as a disease process that is progressive in nature. Doing so enables the clinician to perform diagnostic testing on the diseases that are more likely to occur.*

**MRI of the brain was within normal limits. An MRI of the lumbar spine and bilateral hips showed lumbar degenerative disease with spinal stenosis at L4-5. No cord compression was appreciated. A diffuse signal abnormality throughout all visualized musculature was noted. These areas had diffuse signal enhancement which were nonspecific.**

**Acetylcholinesterase antibody was negative.**

Another important distinction would be classifying his presentation as asymmetric vs. symmetric disease. The patient’s exam is consistent with bilateral involvement without evidence of UMN or LMN pathology and suggests the disease is localizing to the muscle. Potential causes include drugs (fluroquinolone, statins), endocrine disorders (adrenal insufficiency, Cushing syndrome, thyroid dysfunction), and myopathies. Metabolic derangements such as hypomagnesemia and hypokalemia can cause generalized weakness but are not typically proximal.

Given the patient’s nonspecific but concerning involvement of the musculature on the MRI, one must consider myopathic disease. Polymyalgia rheumatica typically presents with pain and stiffness more than weakness and is usually rapidly responsive to low dose steroid treatment. This diagnosis would be atypical since he has no pain and did not improve with prednisone.^6^ Dermatomyositis can cause proximal muscle weakness; however, the patient has no dermatologic findings to support this (Gottron papules, heliotrope rash, or shawl sign). Polymyositis is another possibility given that no rash is present, but the patient has no findings suggestive of interstitial lung disease or GI symptoms. Creatinine kinase levels would be elevated in both dermatomyositis and polymyositis.

The patient is on a statin which can cause a toxic statin-induced myopathy, typically associated with myalgias. A rarer complication of statins is anti-3-hydroxy-3-methylglutaryl coenzyme A reductase (HMGCR) myopathy, an autoimmune process causing an inflammatory necrotizing myopathy. Anti-HMGCR myopathy can occur at any time, even years after consistent statin use.^7^ The disease process persists despite discontinuation of the statin and can be quite debilitating. It requires immunosuppressive therapies as treatment. Regardless, while the patient is hospitalized, it would be wise to hold the statin to see if his weakness improved. Severe rhabdomyolysis from a statin is also plausible but would expect the patient to have symptoms such as cramping, pain, and possibly oliguria secondary to an acute kidney injury.^7^

**The basic metabolic panel was significant for a BUN of 60 mg/dL and creatinine of 1.26 µmol/L. Magnesium and potassium levels were normal. During this time, the creatinine rose to a peak of 3.76 and his edema worsened. Oliguria was noted.**

**The patient’s urinalysis showed 30 mg/dL protein, 500 leukocytes/µL, negative nitrites, 2 + blood, 50 mg/dL glucose, 3.0 mg/dL urobilinogen, 13 RBC/HPF, 84 WBC/HPF, few bacteria, and 2 hyaline casts per LPF.**

**Hepatic function was normal.** **Complete blood count was significant for 31,000 white blood cells/µL with neutrophilia. No blasts were seen.** **Hemoglobin** **was 11.9 g/dL. Platelets were 302** **×** **10**^**9**^**/L**. **C-reactive protein was elevated at 15.1 mg/dL.** **The sedimentation rate was elevated at 70 mm/h.**

**TSH was normal at 0.49 mIU/L. Cosyntropin testing was normal.**

**Creatine kinase (CK) was low at** **8 u/L, aldolase was normal at 8.6 u/L, and lactate dehydrogenase was normal at 221 u/L. Anti-HMGCR antibody was negative.**

The patient’s pitting edema, elevated BUN, and creatinine point toward a kidney injury which could be collateral damage or part of a primary process. While uncommon, muscle breakdown from inflammatory myopathies can cause a myoglobinuria-induced acute tubular necrosis.^8^ Alternatively, the patient could have developed rhabdomyolysis-induced kidney injury from being bed-bound for several days. The normal CK, aldolase, LDH, and anti-HMGCR antibody however lower the likelihood of inflammatory myopathies. A thyroid-related myopathy is unlikely given a normal TSH.

The elevated white blood cell count, CRP, and ESR point toward a systemic inflammatory response. Prior to admission, the patient was prescribed prednisone, which could explain the rise in white blood cells secondary to neutrophil demargination. Certain leukemias and lymphomas are possibilities, but one would expect B-type symptoms and abnormal cells on the differential.^9^

Occam’s razor and Hickam’s dictum can be helpful clinical reasoning tools to explore. Does the patient have a unifying diagnosis or multiple concurrent diseases? The evaluation thus far has identified numerous findings that are abnormal. Revisiting the patient’s initial problem representation with new data can aid in identifying the proper evaluation, diagnosis, and treatment.

Initial representation: “An 88-year-old man with hypertension presented to the emergency room with six weeks of progressive bilateral lower extremity weakness.”

Updated representation: “An elderly man with a history of uncontrolled hypertension presents with subacute, progressive motor weakness of the lower extremities, worsening pitting edema, hematuria, and acute kidney injury with labs concerning for a systemic inflammatory syndrome.”

Although a biopsy of the muscle would likely be helpful obtaining a diagnosis, the results would not return in time to help with management of the disease process that appears to be rapidly progressing. The patient now has worsening edema, acute kidney injury, and hematuria which raises the concern for glomerulonephritis.^10^

Glomerulonephritis (GN) is a disease process that involves the abrupt onset of hematuria, acute renal failure, and fluid retention (edema and hypertension). Proteinuria and pyuria can also occur. Given the patient’s rapid decline in renal function, rapidly progressive glomerulonephritis (RPGN) should be considered. This rare disorder can occur as a primary disorder or be associated with other primary glomerular diseases. RPGN can also be associated with vasculitis and systemic lupus erythematosus.^10^

Revisiting the patient’s presentation with new data allows one to shift the differential of common neurological and muscular causes of motor weakness to the suspicion of a glomerular disease that could cause motor weakness. Common causes of motor weakness have been excluded and now rare presentations of glomerulonephritis should be considered.

*The clinician has reframed the patient’s problem representation which allows the focus to shift the evaluation and differential from proximal motor weakness and acute kidney injury to glomerulonephritis. The case now has a diagnostic clue of which can suggest a new path forward: glomerulonephritis. This disease process serves as a diagnostic foothold.* ^*11*^ *An analogy to a diagnostic foothold to a clinician is like a base camp for a mountain explorer. Having a logical position to “start” the work up allows one to branch out and explore (or in this case, vasculitis syndromes that can present with glomerulonephritis).*

*By using glomerulonephritis as a diagnostic foothold, the clinician can review the most common forms of glomerulonephritis and compare the patient’s presentation to illness scripts of different types of GN.*

**ANA was positive with a 1:160 titer with an atypical speckled pattern. Perinuclear ANCA was positive with a 1:320 titer. Anti-GBM was negative. Complement levels were normal. HIV, hepatitis B, and hepatitis C tests were negative.** **A muscle biopsy and kidney biopsy were obtained.**

The constellation of symptoms and labs raises the concern of a vasculitis causing glomerulonephritis and myopathy. The patient’s presentation and ANCA positivity suggest that the patient has a small-vessel vasculitis. A literature review revealed case reports of a proximal muscle weakness as the presenting symptoms for an ANCA vasculitis.^12^ Myopathy can be the initial manifestation of vasculitis and is often unrecognized. Most patients present with proximal predominant weakness with normal CK and elevated aldolase levels. Furthermore, certain medications (Table 1) and malignancies are associated with vasculitis. On review of his medications and past medical history, the patient was recently started on hydralazine, which has been associated with ANCA vasculitis. Further immunoassay testing of the positive ANCA can further help delineate the cause as drug induced vs. idiopathic (Table 2).

**Table 1:** Medications Implicated With Drug-Induced ANCA-Associated Vasculitis

Medications implicated with drug-induced ANCA-associated vasculitis
Hydralazine
Propylthiouracil
Thiamazole
Sofosbuvir
Minocycline
Carbimazole
Mirabegron
Nintedanib
D-penicillamine
Allopurinol
Rifampicin
Montelukast
Rosuvastatin

**Table 2:** Testing for Drug-induced Versus Idiopathic ANCA-Associated Vasculitis

	**Drug-induced ANCA-associated vasculitis**	**Idiopathic ANCA-associated vasculitis**
ANCA	Present	Present
Anti-MPO antibody	Common	Can be present
Anti-PR3 antibody	Rare	Can be present
Antiphospholipid antibody	Can be present	Rare
Anti-histone antibody	Can be present	Absent
Immune complexes	Rare	Absent

*The consequence of diagnostic errors in clinical practice has allowed further exploration in reflective practice to reduce these errors and improve diagnostic performance.* ^*13*^ *Schön linked reflection to improve professional development and professional practice.* ^*13*^ *In clinical practice, deliberate reflection substantially increased accuracy on complex cases relative to intuitive reasoning.* ^*13*^ *In complex or atypical cases, clinicians may not have a history or physical exam consistent with the disease process at hand. In this case, incorporating new data as it is obtained into the diagnostic reasoning process is paramount in high morbidity and mortality cases. The clinician reflected on the problem representation throughout the case — going through a vast differential of causes of proximal motor weakness and reframed the patient’s problem as an inflammatory syndrome causing acute kidney injury. The clinician ultimately found a diagnostic foothold through guided reflection to obtain a diagnosis that fits the patient’s presentation (suspected drug-induced vasculitis).*

**Further immunoassay testing of the positive ANCA was performed. Anti-myeloperoxidase antibodies (MPO) were elevated at 17.2 units and anti-histone antibodies were elevated to 2.4 units.** **PR-3 antibody was not detected.**

**The muscle biopsy revealed skeletal muscle with focal vessels showing transmural inflammation consistent with vasculitis (Fig. **1**, Fig.** 2**). A subsequent kidney biopsy was unremarkable for acute pathology; however, the sample was limited.**

**Figure 1:**
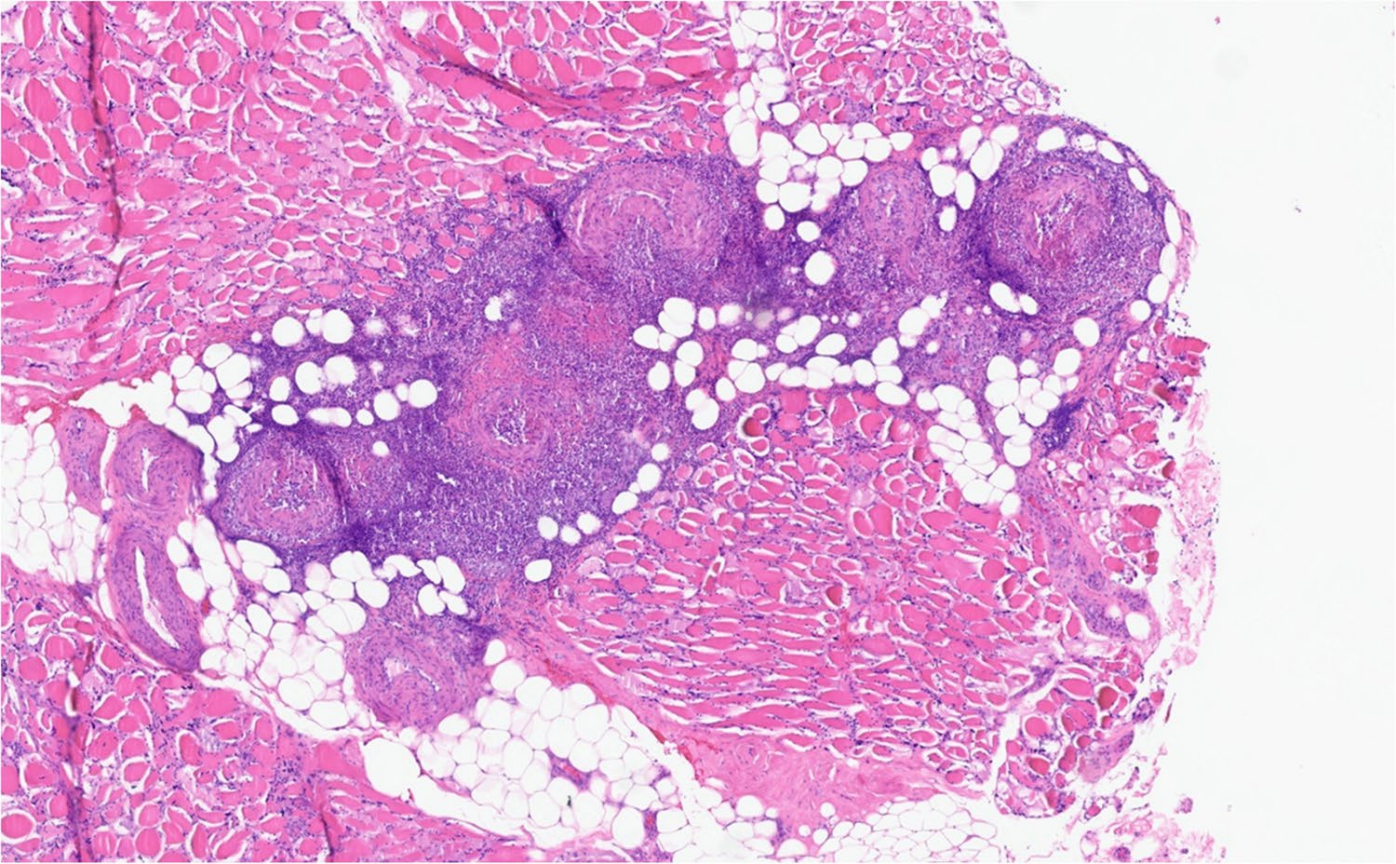
Paraffin H&E stain of the left rectus femoris muscle showing extensive transmural inflammation consistent with active necrotizing vasculitis (4 ×).

**Figure 2:**
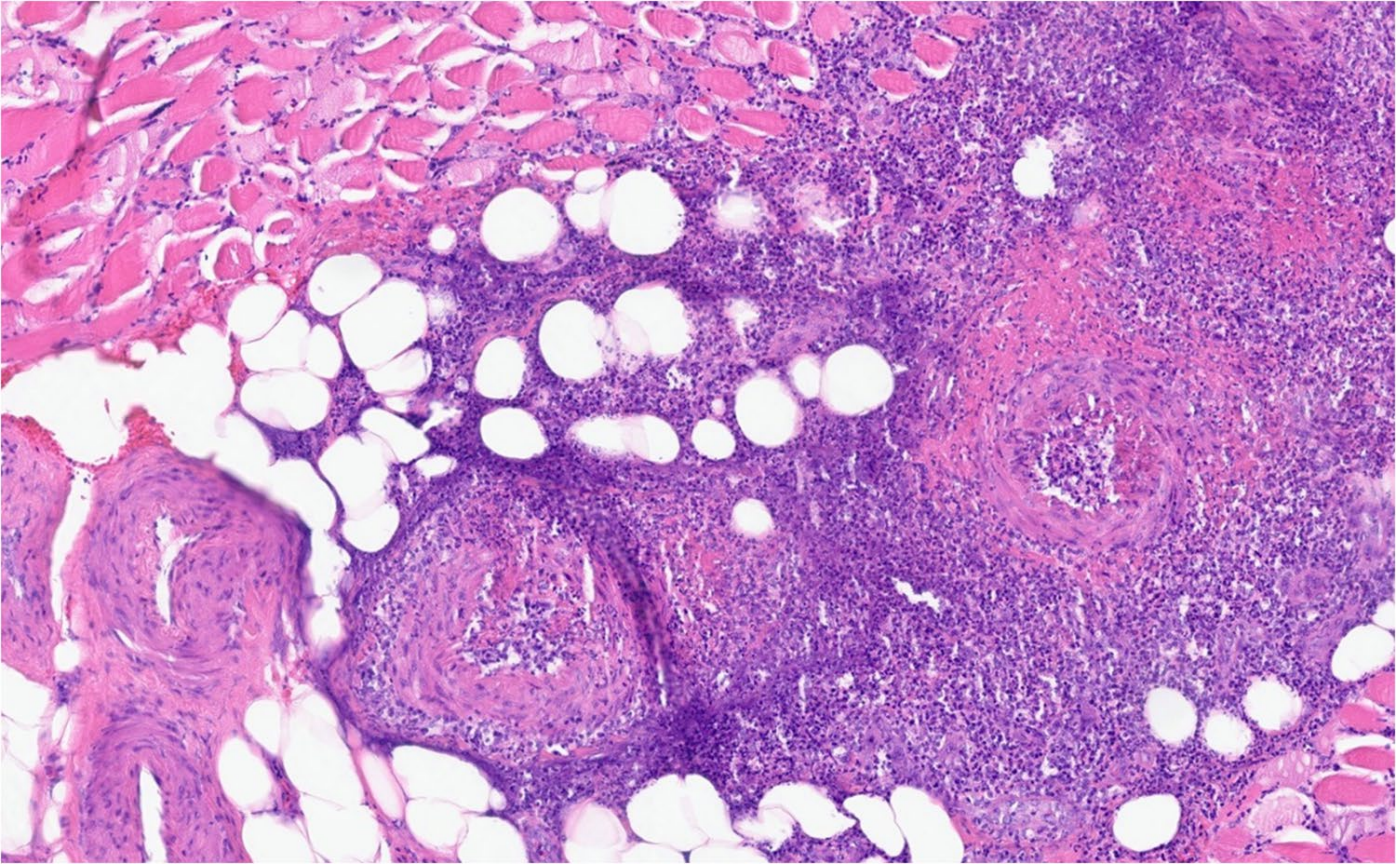
Paraffin H&E stain of the left rectus femoris muscle showing extensive transmural inflammation consistent with active necrotizing vasculitis (10 ×).

**He was immediately taken off hydralazine. Pulse dose steroids were started at 125 mg IV for 3 days followed by 60 mg IV daily. Rituximab was also initiated. His proximal muscle weakness and kidney function improved. Steroids were tapered over several weeks.**

## DISCUSSION

The positive ANA and P-ANCA coupled with a muscle biopsy showing vasculitis confirmed the diagnosis of ANCA-associated vasculitis (AAV). Anti-myeloperoxidase and anti-histone antibodies were ordered to further confirm and classify the vasculitis as drug induced. Most cases of drug-induced AAV have P-ANCA and MPO positivity. Anti-histone antibodies are more commonly found in drug-induced AAV (as found in this case). Drug-induced AAV often presents with nonspecific symptoms such as fever and malaise and can include lung but more commonly kidney involvement. Proximal lower extremity weakness as a presenting symptom is extremely rare.

This complicated case highlights the importance of utilizing a diagnostic framework, reframing the presenting illness, finding a diagnostic foothold, and considering unusual presentations of disease with reflective practice. The discussant first used a diagnostic framework of lower extremity weakness to help localize the disease process. For learners, employing a framework to the presenting problem can help them effectively participate in patient evaluations. Furthermore, the discussant’s revisiting of the problem representation with new data helped refocus the clinical evaluation. As the diagnostic process evolved, the patient’s inflammatory labs (ESR, CRP) coupled with worsening acute kidney injury and hematuria allowed the discussant to expand the differential to vasculitis syndromes. This was particularly important in this case given the morbidity and mortality risks with RPGN. Reframing the problem when the patient’s renal function declined helped the discussant find a diagnostic foothold (glomerulonephritis) which led to a tissue and serologic diagnosis.

### Clinical Teaching Points

1. Vasculitis can present in unusual ways, and this case is one of the few documented cases of ANCA vasculitis presenting with isolated proximal muscle weakness.^12^ In patients with similar symptoms, testing for ANCA is recommended followed by MPO and PR-3 antibody testing (Table 2).
2. Hydralazine is a known precipitant of ANCA vasculitis. Initiation of the medication in patients with a history of autoimmune disease could present significant risks. Symptoms may begin months to years after starting the medication, with one study showing an average of 22 months.^14^ Propylthiouracil, penicillin, cephalosporins, minocycline, allopurinol, D-penicillamine, and sulfasalazine are also causative agents (Table 1).^15^
3. Tissue biopsy is recommended to confirm the diagnosis.

## References

[1] Boehme AK, Esenwa C, Elkind MS (2017). Stroke Risk Factors, Genetics, and Prevention. Circ Res..

[2] Larson ST, Wilbur J (2020). Muscle Weakness in Adults: Evaluation and Differential Diagnosis. Am Fam Physician..

[3] Yew KS, Cheng EM (2015). Diagnosis of acute stroke. Am Fam Physician..

[4] Dreicer J, Parsons AS, Joudi T (2023). Perspect. Med Educ..

[5] Larson ST, Wilbur J (2020). Muscle Weakness in Adults: Evaluation and Differential Diagnosis. Am Fam Physician..

[6] Nesher G (2014). Polymyalgia rheumatica–diagnosis and classification. J Autoimmun..

[7] Tomaszewski M, Stępień KM, Tomaszewska J, Czuczwar SJ (2011). Statin-induced myopathies. Pharmacol Rep..

[8] Couvrat-Desvergnes G, Masseau A, Benveniste O (2014). The spectrum of renal involvement in patients with inflammatory myopathies. Medicine (Baltimore)..

[9] Kameda G, Vieker S, Duck C, Blaes F, Längler A (2010). Paraneoplastic myopathy as a very rare manifestation of acute lymphoblastic leukemia. Klin Padiatr..

[10] **Naik RH, Shawar SH. **Rapidly Progressive Glomerulonephritis. [Updated 2023 May 1]. In: StatPearls [Internet]. Treasure Island (FL): StatPearls Publishing; 2024 Jan-. Available from: https://www.ncbi.nlm.nih.gov/books/NBK557430/32491362

[11] May JE, Blackburn RJ, Centor RM (2018). Pivot and Cluster: An Exercise in Clinical Reasoning. J Gen Intern Med..

[12] Nagiah S, Saranapala DMM (2020). Severe proximal muscle weakness with normal CK as a presenting feature of ANCA-associated vasculitis. BMJ Case Rep..

[13] Mamede S, Schmidt HG (2023). Deliberate reflection and clinical reasoning: Founding ideas and empirical findings. Med Educ..

[14] Kumar B, Strouse J, Swee M (2018). Hydralazine-associated vasculitis: Overlapping features of drug-induced lupus and vasculitis. Semin Arthritis Rheum..

[15] Radić M, Martinović Kaliterna D, Radić J (2012). Drug-induced vasculitis: a clinical and pathological review. Neth J Med..

